# Supplementary figures and images for: Characterization of Poldip2 knockout mice: Avoiding incorrect gene targeting
Source: PLoS One. 2021 Dec 20;16(12):e0247261. doi: 10.1371/journal.pone.0247261 (PMC8687530; doi:10.1371/journal.pone.0247261)

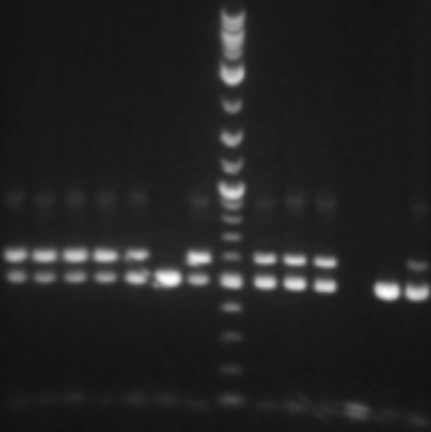

Supplement: S1 File — (ZIP) [file pone.0247261.s001.zip › Original blots & gels/Fig 2B left.pdf]

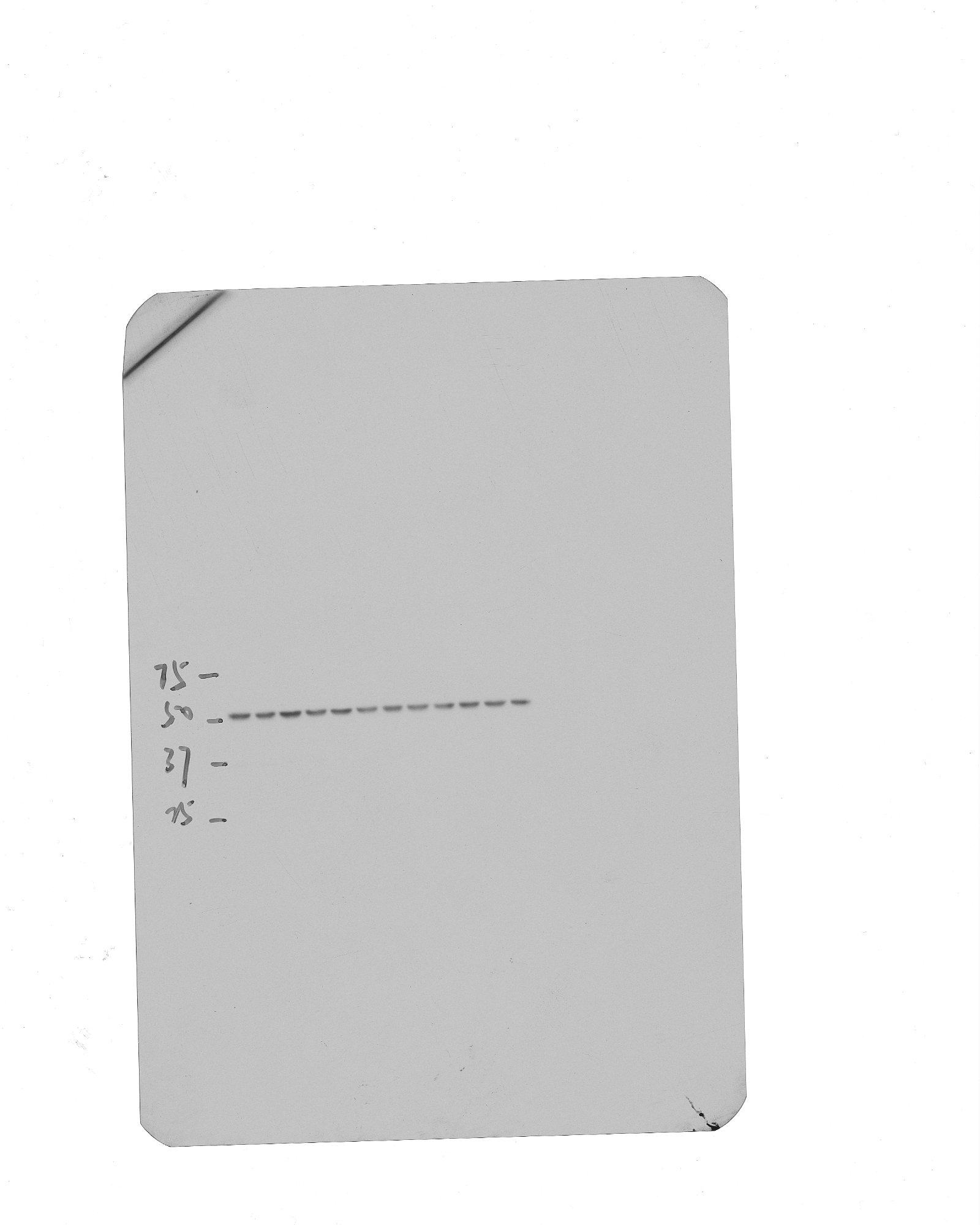

Supplement: S1 File — (ZIP) [file pone.0247261.s001.zip › Original blots & gels/Fig 7B lower blot.jpg]

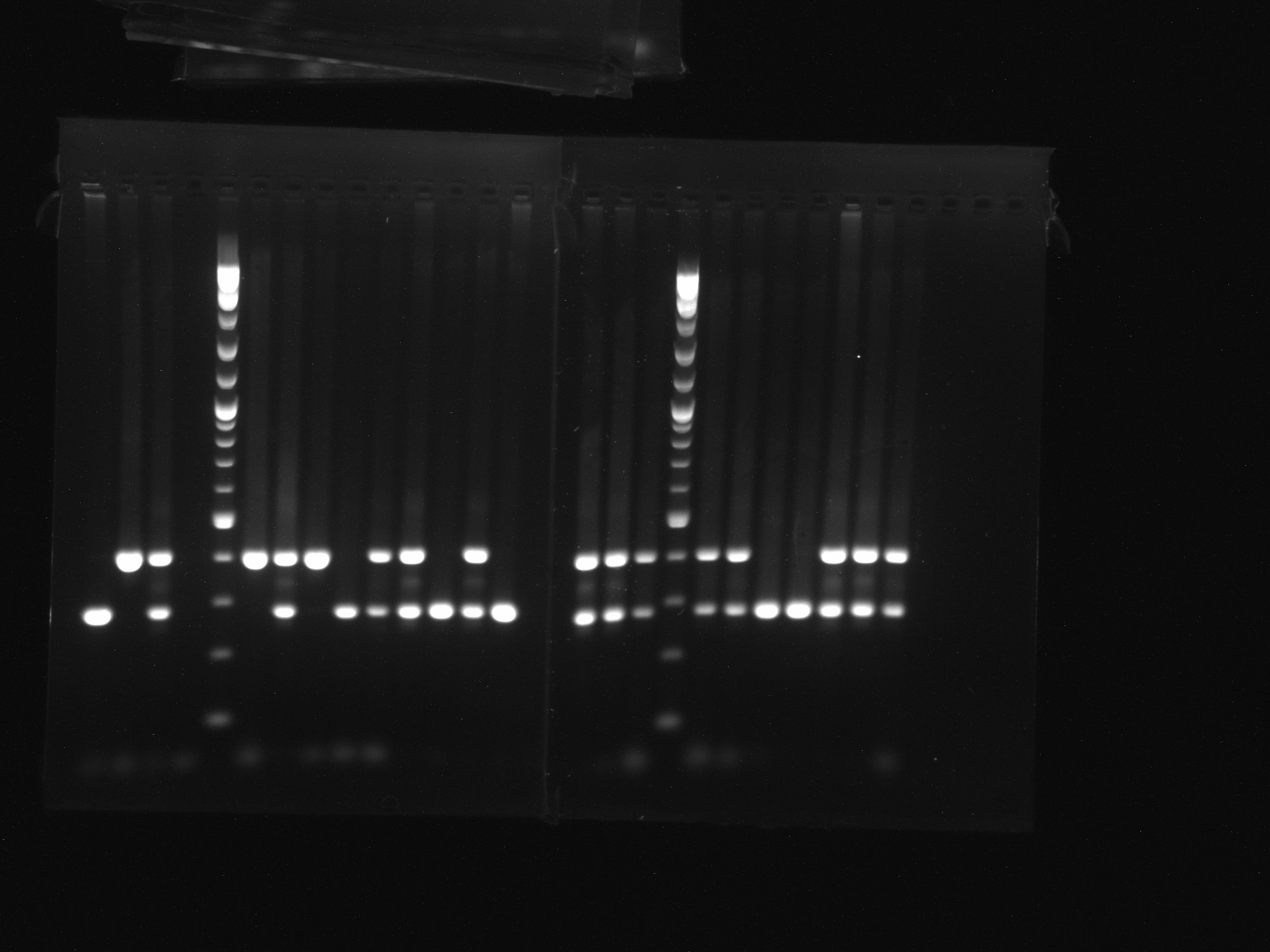

Supplement: S1 File — (ZIP) [file pone.0247261.s001.zip › Original blots & gels/Fig 6B.JPG]

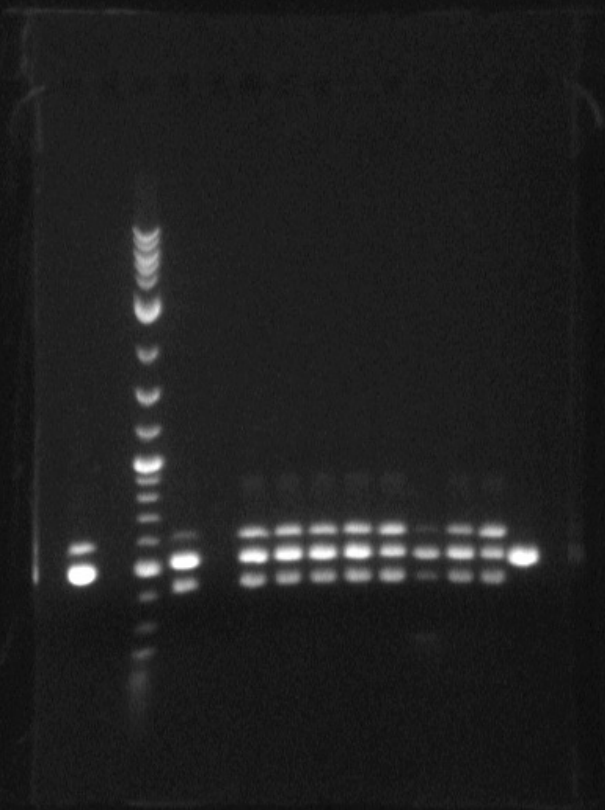

Supplement: S1 File — (ZIP) [file pone.0247261.s001.zip › Original blots & gels/Fig 5C right.pdf]

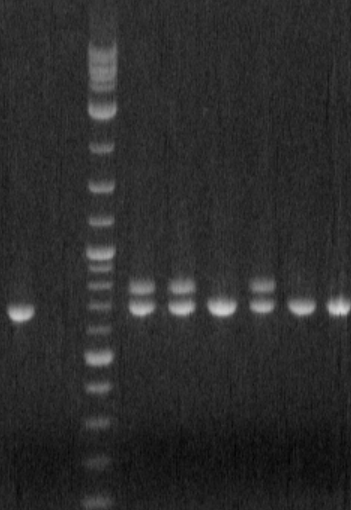

Supplement: S1 File — (ZIP) [file pone.0247261.s001.zip › Original blots & gels/Fig 2B right.pdf]

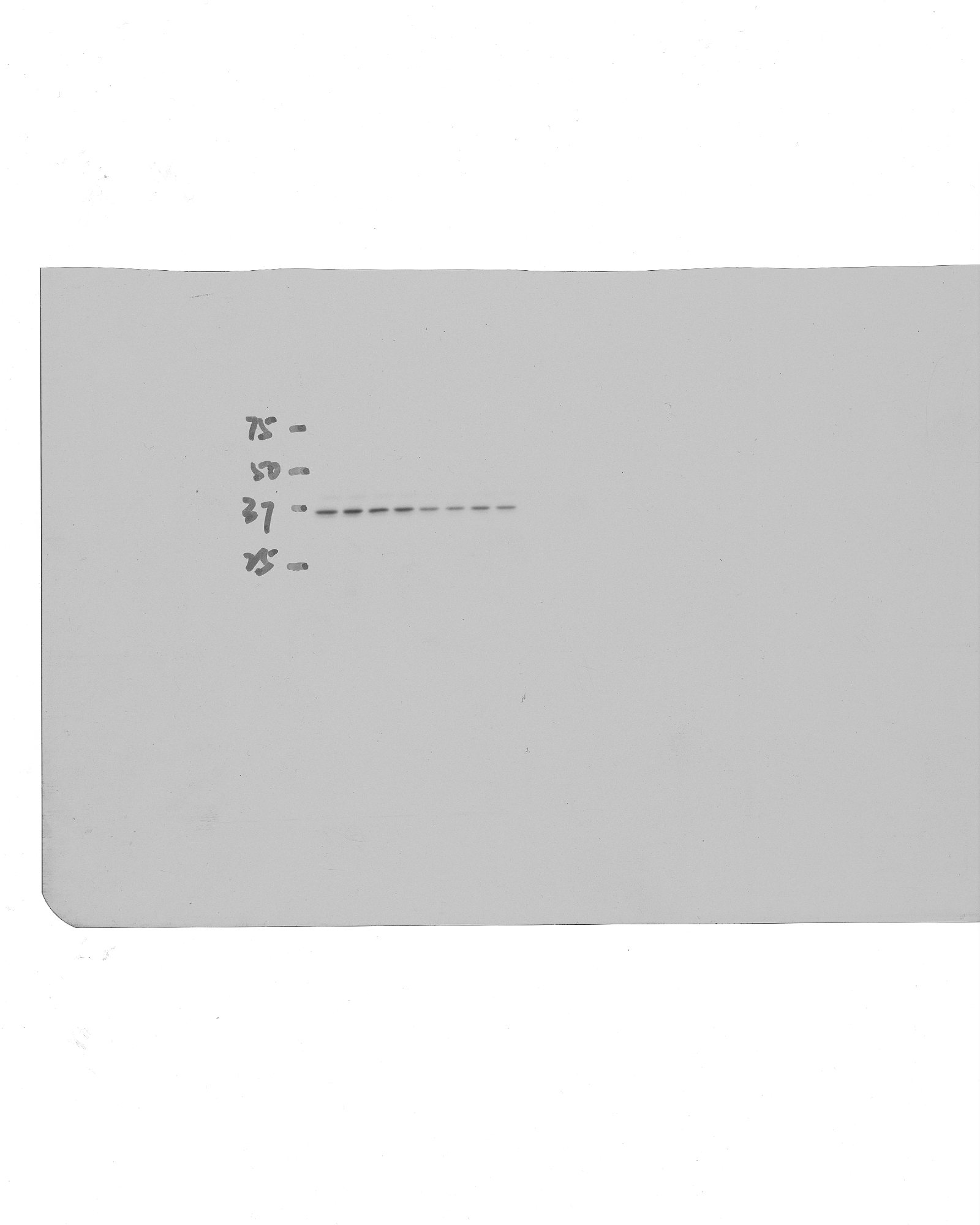

Supplement: S1 File — (ZIP) [file pone.0247261.s001.zip › Original blots & gels/Fig 7B upper blot.jpg]

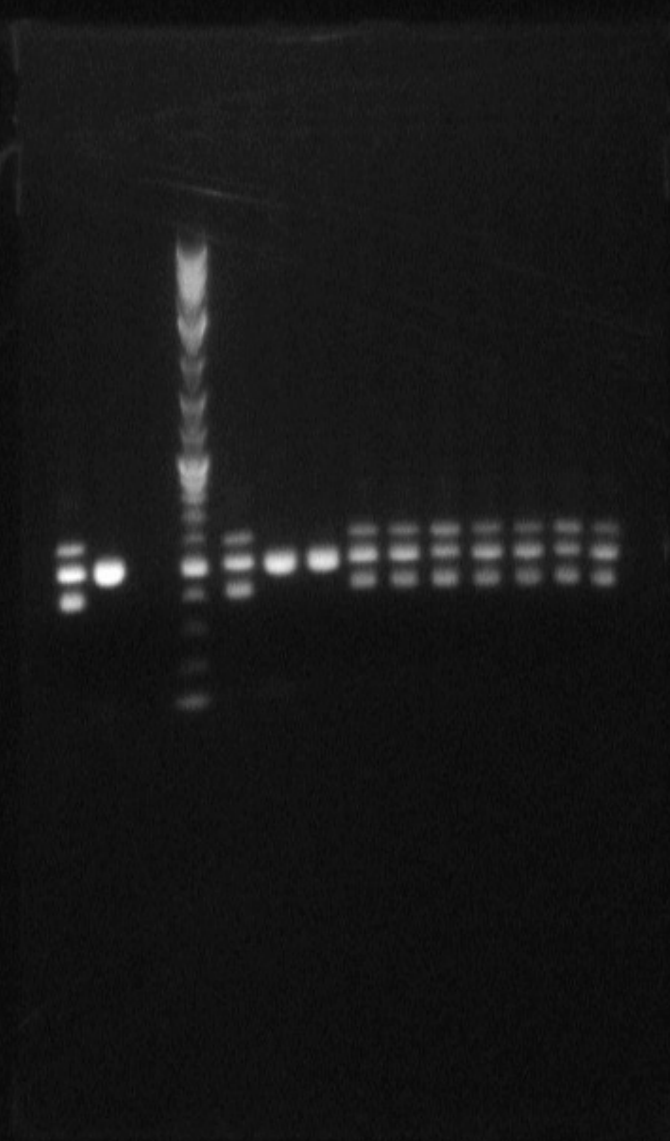

Supplement: S1 File — (ZIP) [file pone.0247261.s001.zip › Original blots & gels/Fig 5C left.pdf]

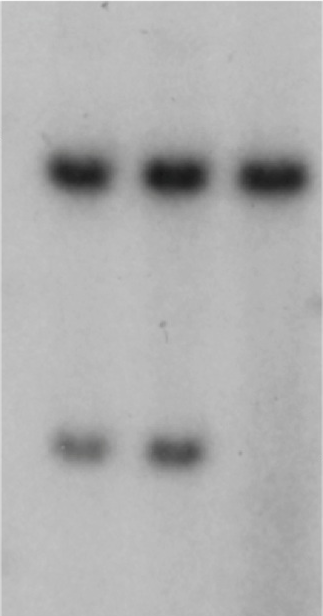

Supplement: S1 File — (ZIP) [file pone.0247261.s001.zip › Original blots & gels/Fig 1B right.png]

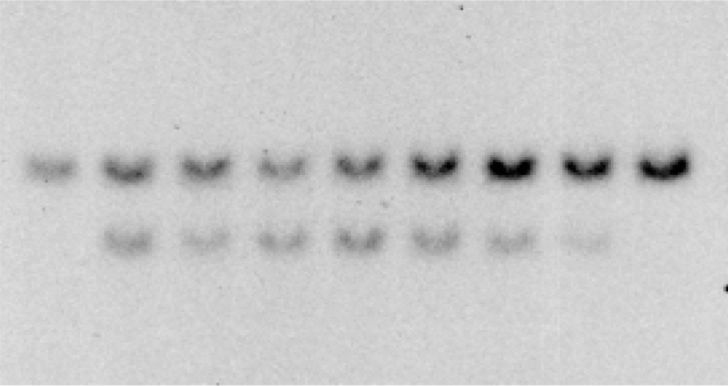

Supplement: S1 File — (ZIP) [file pone.0247261.s001.zip › Original blots & gels/Fig 1B left.png]
